# Genetic characteristics and prognostic implications of m1A regulators in pancreatic cancer

**DOI:** 10.1042/BSR20210337

**Published:** 2021-04-09

**Authors:** Qingyuan Zheng, Xiao Yu, Qiyao Zhang, Yuting He, Wenzhi Guo

**Affiliations:** 1 Department of Hepatobiliary and Pancreatic Surgery, The First Affiliated Hospital of Zhengzhou University, Zhengzhou 450052, China; 2 Key Laboratory of Hepatobiliary and Pancreatic Surgery and Digestive Organ Transplantation of Henan Province, The First Affiliated Hospital of Zhengzhou University, Zhengzhou 450052, China; 3 Open and Key Laboratory of Hepatobiliary and Pancreatic Surgery and Digestive Organ Transplantation at Henan Universities, Zhengzhou 450052, China; 4 Henan Key Laboratory of Digestive Organ Transplantation, Zhengzhou 450052, China

**Keywords:** N1-methyladenosine, Pancreatic cancer, Prognostic marker, RNA modification

## Abstract

Studies have identified the methylation of N1 adenosine (m1A), an RNA modification, playing an important role in the progression of the tumorigenesis. The present study aimed to analyze the genetic characteristics and prognostic value of m1A regulators in pancreatic cancer. In the present study, data on gene mutations, single-nucleotide variants (SNVs), and copy number variation (CNV) were obtained from 363 patients with pancreatic cancer in the Cancer Genome Atlas (TCGA) database, and survival analysis was performed using the logarithmic rank test and Cox regression model. The chi-squared test was used to examine the relationship between the changes in m1A regulatory factors and clinicopathological characteristics. And we used ICGC database to verify the reliability of prognostic markers. The results show that changes in m1A-regulating genes are related to clinical stage and that the expression of some m1A-regulating genes is positively correlated with CNV. In addition, the low expression of the ‘eraser’ gene ALKBH1 is related to the poor prognosis of patients with pancreatic cancer, and its expression level has important clinical significance for patients with pancreatic adenocarcinoma (PAAD). Mechanistically, ALKBH1 may participate in the occurrence and development of pancreatic cancer through mTOR and ErbB signaling pathway. The expression of m1A-regulating genes can be used as a prognostic marker for pancreatic cancer. These findings provide valuable clues for us to understand the epigenetics of m1A in pancreatic cancer.

## Introduction

Pancreatic adenocarcinoma (PAAD) is a highly malignant tumor of the digestive system. According to statistics from the International Agency for Research on Cancer, PAAD causes approximately 430,000 deaths each year, making it the seventh most common cause of cancer-related death among men and women [1]. PAAD will become the second leading cause of death from malignant tumors globally by 2030 [2,3]. Overall, 80–90% of PAAD cases are ductal adenocarcinoma, which is the most common type of PAAD. The vast majority of patients with pancreatic ductal adenocarcinoma (PDAC) have locally advanced or distant metastatic disease (80–85%), only a small proportion of patients are eligible for surgical resection (15–20%), and the 5-year survival rate is less than 6% [4–6]. Surgery is still the main treatment for pancreatic cancer, and the development of chemotherapy and immunotherapy in the oncological field has improved the treatment of pancreatic cancer but to little effect. A recent study found that neoadjuvant chemotherapy can significantly improve the survival of patients with PDAC [7,8]. However, generally speaking, more effective treatments to reduce the recurrence, progression and death of pancreatic cancer need to be further developed. Therefore, it is necessary to explore the molecular mechanisms and pathogenesis of pancreatic cancer and to find new, precise, and individualized therapeutic targets.

Methylation has been extensively studied in tumor progression, it can occur at almost all nitrogen positions of bases. An abnormal increase or decrease in DNA methylation may be a sign of cancer formation and tumor progression [9]. There are multiple mechanisms of interference between epigenetic and metabolic pathways in pancreatic cancer, which ultimately contribute to cell plasticity and tumorigenesis. Epigenetic modification of DNA and histones functions primarily at the transcriptional level, methylation is more helpful in providing new and accurate treatment strategies for the early diagnosis and prognostic prediction of pancreatic cancer [10,11]. In recent years, the role of RNA in cells has also attracted extensive attention and research. There are more than 100 kinds of posttranscriptional modifications of RNA molecules: methylation is the most common, which mainly regulates gene expression at the post-transcriptional level. The most common RNA methylation modifications are methylation of N6 adenosine (m6A) and uridine modifications [12]. It has been found that m6A is related to the occurrence and development of many tumors. For example, there is a significant relationship between changes in gene markers and clinical characteristics in the progression of clear cell renal cell carcinoma [13]. In addition, there has also been increased interest in the study of the RNA methylation modifications N1 methyladenosine (m1A) [14] and 5-methylcytosine (m5C) [15].

m1A is an important posttranscriptional modification of RNA that was first isolated from RNA by Dunn [16]. Under the action of a methyltransferase, m1A can be formed by adding a methyl group to the N1 position of adenosine. The m1A status is mediated by a set of genes: ‘writers’ (TRMT10C, TRMT61B, TRMT6/61A), ‘readers’ (YTHDF1, YTHDF2, YTHDF3, and YTHDC1), and ‘erasers’ (ALKBH1, ALKBH3) [17–19]. m1A methyltransferase has been extensively studied since m1A was first found in tRNA. In terms of the ‘writers’, they are methyltransferases that manipulate the level of m1A to interfere with translation. Human mt-tRNAs are known to contain m1A at positions 9 and 58. TRMT61B and TRMT6/61A catalyse m1A at position 58 of mt and cyt tRNA in human cells, and TRMT10C catalyses it at position 9 [20–22]. The ‘readers’ read the information of methylation modification and participate in the translation and degradation of downstream RNA. Proteins containing YTH domains, namely, YTHDF1, YTHDF2, YTHDF3, and YTHDC1, directly bind to readings of RNA with m1A [19]. The ‘erasers’ are demethylases that catalyse the demethylation of m1A single-stranded DNA and RNA [18]. Two AlkB family proteins, ALKBH3 and ALKBH1, have been found to demethylate m1A. A study reported that ALKBH3 can function as a tRNA demethylase to promote protein synthesis in cancer cells [23].

With the development of high-throughput sequencing technology, it has been found that m1A is widely present in tRNA, rRNA, and mRNA, which participates in the occurrence and progression of many diseases. M1A modification is involved in a variety of cellular processes, leading to reduced cell viability, impaired self-renewal ability, developmental defects, and cell death. It has been reported that m1A modification clearly enhances the process of protein expression and that it is also ubiquitous in humans, rodents, and yeast [24]. Recently, changes in m1A-regulating genes have been shown to promote the progression of bladder cancer [25]. At present, we know very little about m1A in the occurrence and development of cancer. Therefore, in the present study, we analyzed the clinical and pathological data of the pancreatic cancer cohort from the TCGA, evaluated the alterations in the profiles of ten m1A-regulating genes, and explored and speculated about the relationship between m1A-modified gene regulation, tumor mechanisms, and clinicopathological characteristics such as prognosis in pancreatic cancer based on genetic changes and dysfunction.

## Materials and methods

### Data processing

All 363 PAAD data, including mutation, CNV, mRNA expression and clinical data are download from the TCGA website (https://www.cancer.gov/tcga), the download time is September 2019.We obtained 175 SNV data (level 3 data obtained by using MuTect) [26], 183 CNV data, 177 transcriptome data (forms of TPM and FPKM) [27] and 185 cases with clinical information.

For CNV, we divided the cohort of patients with pancreatic cancer into two subgroups, namely, ‘mutations and/or CNV in 10 m1A-regulating genes’ and ‘no mutations or CNV,’ to analyze the clinicopathological significance of CNV and/or mutation status. The mRNA expression data were calculated from RNA-Seq V2 RSEM data; we analyzed the differentially expressed genes in PAAD and normal tissue samples, and then the relationship between mRNA expression and CNV was analyzed.

After integrating the data and excluding the samples with incomplete clinical information and survival time of less than 90 days, 166 samples were used for survival analysis.

### Gene Set Enrichment Analysis (GSEA)

In the present study, according to the level of ALKBH1 gene expression, the patients were divided into two groups, which were incorporated into the GSEA process. Java analysis software was provided by and downloaded from the following website: http://software.broadinstitute.org. We calculated an enrichment score (ES) that reflects the degree to which a set S is overrepresented at the extremes (top or bottom) of the entire ranked list L. Then, we estimated the statistical significance (nominal *P* value) of the ES by using an empirical phenotype-based permutation test procedure. We normalized the ES for each gene set to account for the size of the set, yielding a normalized enrichment score (NES). We then controlled the proportion of false positives by calculating the false discovery rate (FDR) corresponding to each NES. Gene sets with standardized *P*<0.05 and false discovery rate <0.25 were considered significantly enriched.

### LASSO analysis

The LASSO method was introduced into the glmnet R software package to identify the important predictors of m1A-regulating genes [28]. This is a commonly used high-dimensional exponential regression method. The aim was to use 10 M1A-regulating genes in the existing data to predict the survival probability and further reduce the number of prognostic markers. The first step was to screen 366 cases with no gene locus deletion in the data. For the screened data, a prediction model was established with survival probability as the survival state and 10 loci as independent variables. Subsequently, we predicted the risk score of each patient with PAAD and used the median value at risk as the cutoff score. We used the Kaplan–Meier curve to predict the survival rate of patients with PAAD. A pathway from KEGG was constructed for m1A regulators and related protein coding gene signatures by using Cytoscape 3.5.1.

### Statistical analysis and data verification

In the present study, all statistical analyses were performed using SPSS 23 (IBM, Chicago, IL, U.S.A.) and GraphPad Prism 6.0 (GraphPad Software, La Jolla, CA, U.S.A.). We used validated data sets to analyze the relationship between ALKBH1 gene expression and patient survival. Based on COX regression analysis, the risk value of the sample was calculated by using ALKBH1 gene expression and risk value. The chi-squared test was used to analyze the relationship between CNV of m1A-regulating genes and clinicopathological characteristics. Kaplan-Meier curves and log-rank tests were used to evaluate the prognostic value of m1A-regulating gene changes. Univariate and multivariate prognostic risk factors were analyzed by Cox regression model to calculate hazard ratio (HR), 95%CI and *P* values. Univariate analysis was carried out first, and factors with *P*≤0.10 were selected into multivariate analysis to explore independent risk factors affecting prognosis. Used Pearson correlation to calculate the correlation between two classes. All statistical results with *P*<0.05 were considered to be significant.

## Results

### Mutation and CNV of m1A-regulating genes in patients with pancreatic cancer

**Figure 1:**
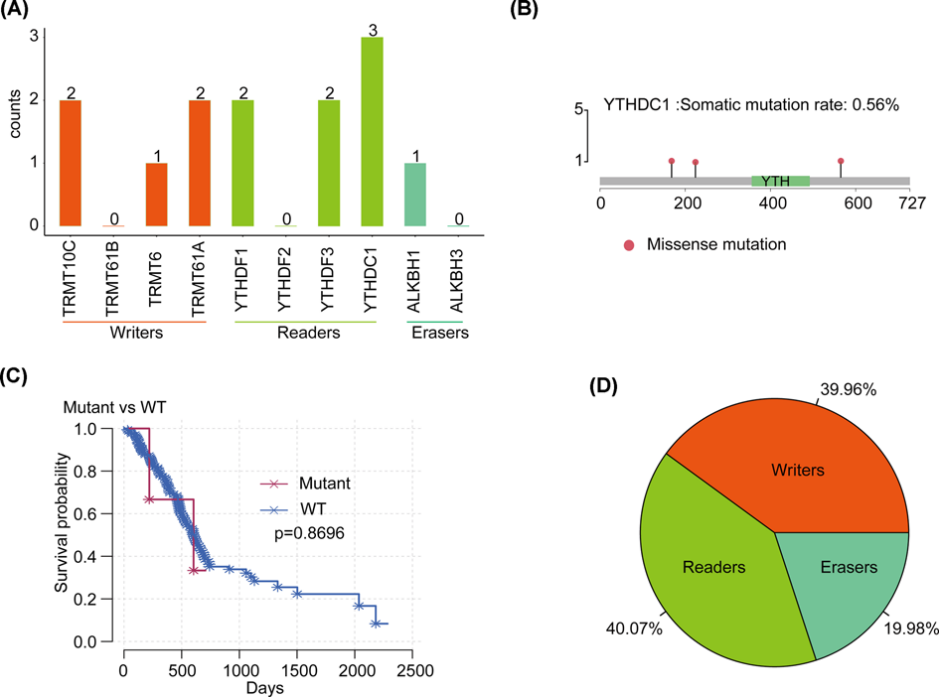
Mutations and CNVs of m1A-regulating genes in PAAD (**A**) Statistics of mutation frequencies of m1A-regulating genes with different functions in PAAD. (**B**) The mutation site of YTHDC1. (**C**) The relationship between eight functionally altered genes and patient survival. (**D**) CNV statistics of m1A-regulating genes in PAAD.

Among 363 cases with sequencing data, we found that m1A regulatory gene mutations occurred in four independent samples (Table 1 and Table 2). Among them, the frequency of mutations of the reader gene YTHDC1 was high, which was detected three times in one sample; counterparts of the reader gene YTHDF1 and the writer gene TRMT61A and TRMT10C were found two times. Particularly, YTHDF1 and TRMT61A appeared in two samples, while the mutation frequency of the eraser gene was relatively low (Figure 1A). In terms of the function of SNV, annotated functional changes occurred in three samples, which are missense mutations in eight m1A-regulating genes except YTHDF2 and ALKBH3. As a reader gene with highest mutation fraction, missense mutation of YTHDC1 may affect the transmission of m1A signals in the cell, leading to dysfunction (Figure 1B). The higher rate of mutations of YTHDC1 in tumor cells suggests a potentially abnormal function of m1A. Besides, we used eight genes with functional changes to predict the survival of PAAD patients and found that the prognosis associated with mutations in eight genes was worse than that in cases without mutations. However, owing to the small number of mutations, the *P* value did not reach the significance threshold (Figure 1C).

**Table 1:** SNV statistics of m1A regulatory genes in PAAD samples

	Writer				Reader				Eraser	
Sample ID	TRMT10C	TRMT61B	TRMT6	TRMT61A	YTHDF1	YTHDF2	YTHDF3	YTHDC1	ALKBH1	ALKBH3
TCGA-IB-7651	p.A128S;p.A196A		p.G59D	p.R83H	p.H101H			p.K565N; p.E224K; p.Q168H	p.A205T	
TCGA-HZ-8001							p.L307*			
TCGA-IB-A5SQ					p.R404C					
TCGA-HZ-7918				p.Y88Y						

In 183 PAAD samples with CNV data, m1A-regulating genes, including writer, reader, and eraser genes, were observed to have a high frequency of CNV at 39.96%, 40.07%, and 19.98%, respectively (Figure 1D). In terms of individuals, the reader gene YTHDF2 had the highest frequency at 21.2%, followed by YTHDF3 at 18.48%. The counterpart of the eraser gene ALKBH3 was the lowest at only 7.07%. The high frequency of CNV illustrates the importance of m1A-regulating genes in PAAD.

**Table 2:** Overview of m1A-regulated mutations in PAAD samples

	Writer				Reader				Eraser	
Sample ID	TRMT10C	TRMT61B	TRMT6	TRMT61A	YTHDF1	YTHDF2	YTHDF3	YTHDC1	ALKBH1	ALKBH3
mutation number	2	0	1	2	2	0	2	3	1	0
mutation fraction	15.38%	0.00%	7.69%	15.38%	15.38%	0.00%	15.38%	23.08%	7.69%	0.00%

### Changes in m1A-regulating genes are associated with clinical pathology and molecular characteristics

We next evaluated the relationship between changes (CNV and/or mutations) in m1A-regulating genes and the clinicopathological characteristics of the patients. We used Cox regression analysis to analyze the clinical features. The results showed that survival of patients with PAAD was associated with a higher T stage (the *P* value was the lowest, but not significant; Table 3). Taking the findings together, for SNV or CNV alone or in combination, there was no significant relationship in patient prognosis. Therefore, we speculated that the changes in m1A-regulating genes may be related to changes in other pathogenic molecules associated with PAAD. Since ATM, BRCA1, CDKN2A, and TP53 play important roles in the pathogenesis of PADD [29], we further evaluated whether the variation in m1A-regulating genes is related to the variation in these four genes. As expected, the changes in m1A-regulating genes were significantly associated with changes in ATM. In the actual test results, nearly half of the pancreatic cancer patients with ATM alterations had changes in the m1A regulatory gene. We speculate that m1A-regulating genes may induce the activation of ATM signaling pathway in the progression of pancreatic cancer.

**Table 3:** Cox analysis of clinical characteristics and changes in m1A regulatory genes

Features	Beta	HR (95% CI for HR)	Wald test	*P*-value
Stage T1	0.62	1.9 (0.98–3.5)	3.6	0.056
Grade 1	0.34	1.4 (0.91–2.2)	2.4	0.12
Stage M1	-0.12	0.89 (0.59–1.3)	0.32	0.57
Stage 1	-0.24	0.79 (0.25–2.5)	0.16	0.69
Stage N1	0.0084	1 (0.24–4.2)	0	0.99

### Relationship between CNV and mRNA expression of m1A-regulating genes

**Figure 2:**
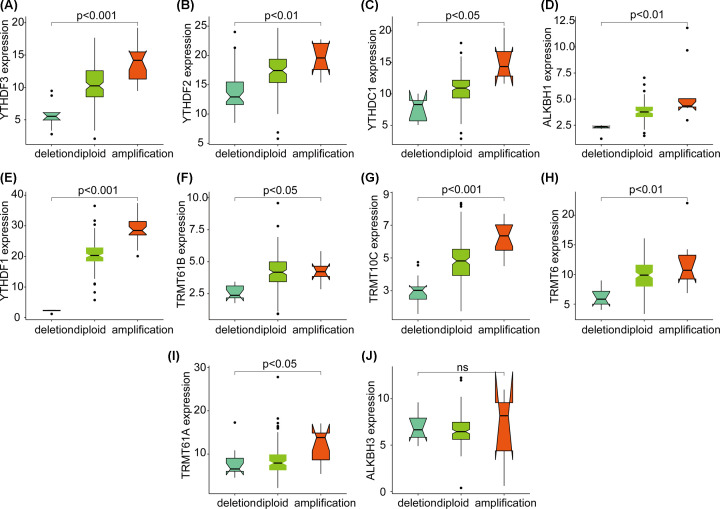
Correlation between the CNV and mRNA expression levels of ten m1A-regulating genes (**A**–**I**) The significant relationship between the CNV and expression levels of nine m1A-regulating genes. (**J**) There was no significant relationship between the CNV and expression level of m1A-regulating genes.

From the above-mentioned findings, the changes in CNV of m1A-regulating genes are more significant than that of SNV, which can affect gene expression levels through a dose compensation effect. As such, we next evaluated the effect of changes in m1A-regulating genes on mRNA expression. The results showed that the expression level of mRNA was significantly correlated with different CNV patterns in 177 PAAD samples. For all 10 regulatory genes, an increase in the copy number of 9 genes was associated with higher mRNA expression; deletions resulted in decreased mRNA expression (Figure 2A–I, all *P*<0.05). These 9 genes are involved in all of the processes of m1A regulation. It is worth noting that the expression of all writer and reader genes is significantly related to CNV, which suggests that CNV of m1A-regulating genes have important regulatory effects.

### Relationship between CNV of m1A-regulating genes and survival of patients with pancreatic cancer

**Figure 3:**
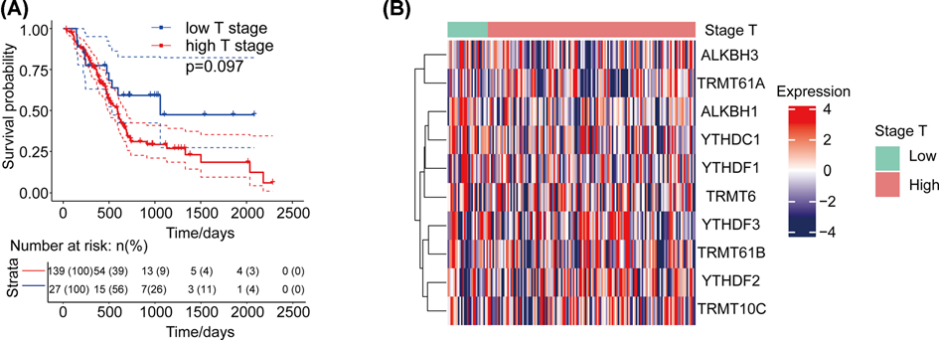
Association between m1A-regulating genes and survival in patients with pancreatic cancer (**A**) The relationship between different clinical stages and patient prognosis. (**B**) Clustering heat map of m1A-regulating genes and cases of different stages.

**Figure 4:**
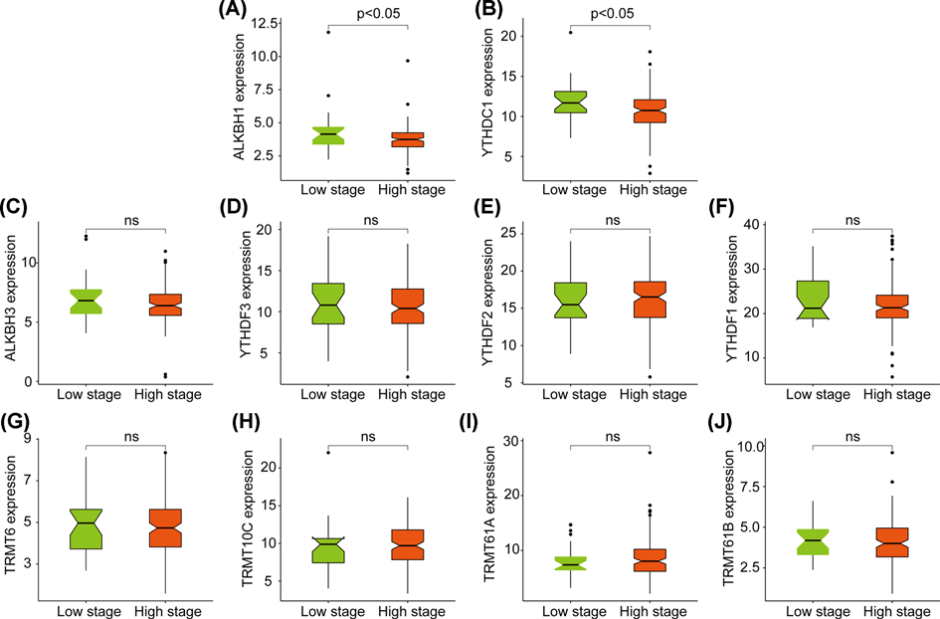
Expression levels of ten m1A-regulating genes in cases of different clinical stages (**A** and **B**) Significant expression of two m1A-regulating genes in cases of different clinical stages. (**C–J**) Eight m1A-regulating genes are not significantly differentially expressed in cases of different clinical stages.

We defined T1/T2 as low-stage cases and T3/T4 or above as high-stage cases, and we observed that higher T stage was related to the prognosis of patients with PADD. High-stage patients had poor survival, but the association was not statistically significant (Figure 3A). Kaplan–Meier survival curves were consistent with the aforementioned T stage results. Based on this classification, we clustered the expression of m1A-regulating genes in different T-stage cases (Figure 3B). Then, we analyzed the differences in the expression of m1A-regulating genes in cases of different clinical stages. The results showed that the expression of ALKBH1 and YTHDC1 was negatively correlated with patients (Figure 4A,B, all *P*<0.05). The expression of the other eight genes was not differently significant (Figure 4C–J, all *P*>0.05). Among the genes, YTHDC1 and ALKBH1 not only had a high mutation rate but also had a positive correlation with CNV at the expression level. This suggests that the expression of m1A-regulating genes is related to the prognosis of patients.

### m1A-regulating genes as prognostic markers of pancreatic cancer

**Figure 5:**
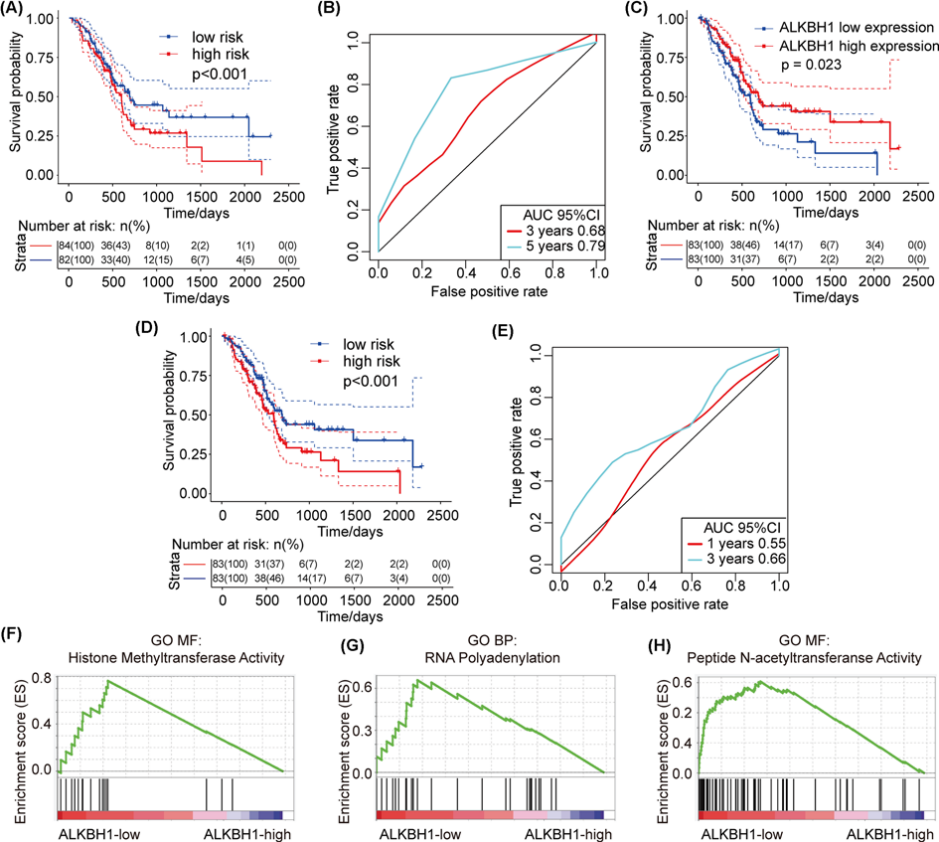
Survival and AUC in PAAD patients and GSEA results of different expression levels of ALKBH1 (**A**) Survival curve of multivariate Cox regression. (**B**) AUC curve of multivariate Cox regression. (**C**) The relationship between ALKBH1 expression and patient prognosis. (**D**) Risk analysis of the ALKBH1 gene. (**E**) AUC of the ALKBH1 gene. (**F**–**H**) GSEA results of ALKBH1 expression. (**F**) histone methyltransferase activity. (**G**) RNA polyadenylation. (**H**) peptide N-acetyltransferase activity.

We have demonstrated that changes in the CNV of m1A-regulating genes cause changes in m1A-regulating gene expression levels. Thus, we next analyzed the relationship between the CNV of m1A-regulating genes and patient survival in PAAD cases. The results showed that there was a very strong positive correlation between the expression of some m1A-regulating genes and CNV. For this reason, we used univariate Cox regression analysis to explore the relationship between different m1A regulatory gene expression levels and the prognosis of patients. The results showed that the expression level of the ALKBH1 gene was significantly correlated with the prognosis of patients (*P*<0.05) (Table 4) and with changes in CNV. In addition, we used multivariate Cox regression analysis to explore the impact of 10 m1A-regulating genes on the prognosis of patients. We found that the expression of m1A-regulating genes can effectively predict the risk of patients (Figure 5A, *P*<0.001) and that the area under the curve (AUC) at 3 and 5 years was greater than 0.65 (Figure 5B). This suggests that the expression of m1A-regulating genes can be used as a prognostic marker for pancreatic cancer.

**Table 4:** Univariate Cox regression exploring the relationship between different m1A regulatory gene expression levels and patient prognosis

Features	Beta	HR (95% CI for HR)	Wald test	*P*-value	CNV sig
ALKBH1	-0.2	0.82 (0.68–1)	4	0.045	yes
YTHDF1	-0.04	0.96 (0.92–1)	3.8	0.052	yes
TRMT61A	-0.043	0.96 (0.9–1)	2	0.15	yes
YTHDC1	-0.058	0.94 (0.87–1)	1.9	0.17	yes
TRMT61B	0.099	1.1 (0.95–1.3)	1.6	0.2	yes
ALKBH3	-0.058	0.94 (0.85–1.1)	1.1	0.3	no
YTHDF3	0.031	1 (0.97–1.1)	1.1	0.3	yes
TRMT10C	0.037	1 (0.97–1.1)	1	0.32	yes
TRMT6	-0.031	0.97 (0.84–1.1)	0.17	0.68	yes
YTHDF2	0.0068	1 (0.95–1.1)	0.06	0.81	yes

Next, through 1000 LASSO analysis of 10 m1A-regulating genes, we found only the ALKBH1 gene has a significant effect on expression levels, whose changes of CNV were significant. Further, the results of univariate Cox analysis and expression levels in clinical stage were obviously related (*P*<0.05) (Table 5). The ALKBH1 gene is an eraser gene that participates in important functional regulation in the process of m1A.

**Table 5:** LASSO analysis of m1A regulatory genes

Duplicates	Genes	Functions	Cnv express sig	Stage sig	Survival sig
637	YTHDC1	reader	yes	yes	no
577	ALKBH1	eraser	yes	yes	yes
211	YTHDF1	reader	yes	no	no
200	TRMT10C	writer	yes	no	no
108	ALKBH3	eraser	no	no	no
103	YTHDF3	reader	yes	no	no
87	TRMT61A	writer	yes	no	no
27	TRMT6	writer	yes	no	no
18	TRMT61B	writer	yes	no	no
0	YTHDF2	reader	yes	no	no

Subsequently, we analyzed the risk related to ALKBH1 gene expression in patients. We used the expression of the ALKBH1 gene as an indicator to determine the prognosis of patients. The results showed that low expression of the ALKBH1 gene was related to poor prognosis in patients (*P*=0.023, Figure 5C). We also used the ALKBH1 gene for Cox regression analysis and found that its expression can effectively be used to analyze and predict the survival of PAAD patients; the AUC at 1 and 3 years was greater than 0.55, and the *P*-value of patient risk prediction was less than 0.0001 (Figure 5D,E, all *P*<0.001). The above results suggested that the expression level of the ALKBH1 gene has clinical significance in patients with PAAD and may be used as a prognostic marker for pancreatic cancer.

### Enrichment analysis of ALKBH1 functional expression levels

The ALKBH1 gene is one of the eraser genes during methylation. Given its importance in the methylation process, we then discussed the role of m1A disorders in the pathogenesis of PAAD. We performed gene set enrichment analysis on samples with different levels of ALKBH1 mRNA expression. Gene enrichment analysis showed that high expression of ALKBH1 is related to the physiological process of histone methylation (Figure 5F–H, all *P*<0.05). The activation of histone methylation may be involved in the pre-transcriptional regulatory process and may be used to silence gene expression. As an eraser gene, high expression of ALKBH1 acts on the activation pathway of methylation, which may cause the methylation modification of writer genes to be removed after editing. These findings suggest that the high expression of ALKBH1 is associated with a favorable prognosis in patients.

### Validation and analysis of ALKBH1 as an important targeting molecule of pancreatic cancer in ICGC data sets

**Figure 6:**
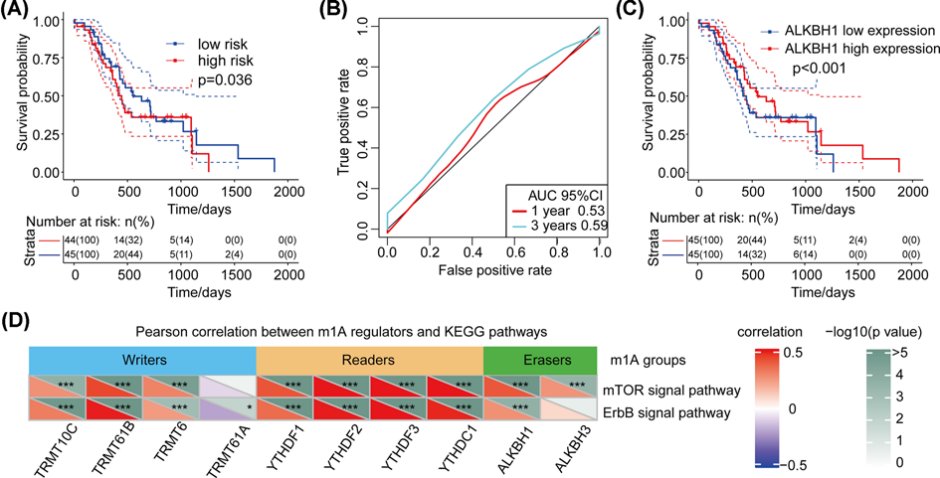
Validation of the relationship between the ALKBH1 gene and survival in the data set (**A**) Risk analysis of the ALKBH1 gene in the validation data set. (**B**) AUC of the ALKBH1 gene in the validation data set. (**C**) The relationship between ALKBH1 expression and patient prognosis. (**D**) Pearson correlation between m1A regulators and KEGG pathways. Relationship of m1A regulator genes with mTOR and ErbB expression. The correlogram shows that major m1A regulator genes have a significant correlation with the mTOR and ErbB signaling pathways. Red represents positive correlations, while blue represents negative correlations; *P<0.05.

Finally, we used validation data sets to analyze the relationship between ALKBH1 gene expression and patient survival. Based on the Cox regression analysis, the risk value of the samples was calculated by using the expression level and risk value of the ALKBH1. We found that the ALKBH1 gene also has good risk prediction ability in the validation data set (ICGC-PACA-AU). The AUC values at 1 and 3 years were near 0.55 (Figure 6A,B, *P*<0.05). In addition, in the ICGC data set, low expression of the ALKBH1 gene was also associated with poor prognosis (*P*<0.001, Figure 6C).

### Signaling pathway regulated by m1A modification

We incorporated m1A-regulating genes into KEGG pathway analysis to verify the activation of related signaling pathways. We analyzed the Pearson correlation between m1A regulators and KEGG pathways through the functional similarity of biological processes such as metabolism, signal transduction, molecular function and cell composition. The greater the absolute value of the correlation coefficient is, the stronger the correlation is. Red represents positive correlations, while blue represents negative correlations. We found that these regulatory genes are mainly involved in 24 signaling pathways, especially mTOR and ErbB. The results showed that all regulatory genes except TRMT61A were significantly associated with the mTOR pathway. Furthermore, there is a strong correlation between eraser ALKBH1 and mTOR and ErbB signaling pathways. This suggests that m1A-regulating genes may be involved in the activation or regulation of the two signaling pathways (Figure 6D).

## Discussion

In the present study, we conducted a retrospective analysis of 363 patients with pancreatic cancer using the TCGA database to study the gene changes and prognostic value of m1A-regulating genes in pancreatic cancer. By analyzing the mutations of the writer, eraser, and reader genes among m1A-regulating genes in pancreatic cancer and the relationship between CNV and the clinical stage and survival time in pancreatic cancer, we have provided a reference for studying the role and prognosis of m1A in pancreatic cancer.

To date, more than 100 different kinds of posttranscriptional modifications have been reported [30]. RNA modification has become a hot research topic, and dynamic modification represents a new direction of epigenetic research. N1 methyladenosine (m1A) is an important posttranscriptional modification of RNA that was first identified more than 50 years ago [16,19]. M1A is the methylation modification on the first nitrogen atom of adenine of RNA molecule. Previous studies have shown that m1A is a post-transcriptional modification with a high abundance in tRNA and rRNA in eukaryotes, and recent studies have also shown that m1A modification can regulate mRNA translation. The methyl group on m1A in mRNA blocks Watson–Crick base pairing, affecting reverse transcription and protein translation [17,31,32]. Changes of CNV in m1A-relating genes regulate mRNA expression levels, modifying protein levels after transcription, to affect the occurrence and development of diseases. For example, another study found that m1A-regulating genes regulate the PI3K/AKT/mTOR and ErbB pathways in gastrointestinal malignancies, demonstrating for the first time the imbalance and signal transduction pathways of m1A in gastrointestinal cancers. In that study, they used TCGA data from five digestive system neoplasms to study the characteristics of nine m1A-relating genes (TRMT61A, TRMT6, TRMT10C, ALKBH1, ALKBH3, YTHDF1, YTHDF2, YTHDF3, and YTHDC1). The results showed that overexpression of ALKBH1 was negatively correlated with the overall survival of stomach adenocarcinoma, colon adenocarcinoma, and liver hepatocellular carcinoma, while low expression of ALKBH3 in esophageal carcinoma and colon adenocarcinoma was related to poor overall survival [33]. Therefore, we speculated that the genes may differ in tumors, providing clues for the study of pancreatic cancer. Especially the prognostic model of m1A in liver cancer has been established [14]. As far as our research is concerned, the fact is that the overexpression of ALKBH1 is negatively correlated with the clinical stage of pancreatic cancer and is associated with a good prognosis.

Furthermore, we found that the frequencies of reader and writer mutations and CNV were higher, indicating that m1A may play other important role in pancreatic cancer. In addition, alterations of the reader gene YTHDF1 and the writer gene TRMT61A were simultaneously present in two samples. We speculated that the m1A writer gene and the reader gene may play a synergistic role in the modification of m1A in RNA. However, we have not yet found a specific function in pancreatic cancer. We studied the role of m6A and m5C methylation in tumors. For example, the high expression of YTHDF1 and YTHDF2 was related to the poor prognosis of patients with hepatocellular carcinoma, indicating that it promotes proliferation and inhibits invasion and metastasis. This has also been reported in pancreatic cancer [34,35]. Therefore, we speculated that the regulation of m1A is also extremely complicated. At the cellular level, different types of tumors have different roles in regulating genes. Guo et al. have found that the deletion of the demethylase ALKBH5 of m6A will aggravate the occurrence of pancreatic cancer and the adverse clinicopathological characteristics [36]. As a new direction of research, further study of the role of the eraser genes of m1A is warranted to explore the regulatory mechanism of m1A in pancreatic cancer.

With regard to the relationship between the changes in m1A-regulating genes and clinicopathological characteristics, we established the existence of a relationship between tumor grade and survival of pancreatic cancer. In analyzing the effects of SNV and CNV on the prognosis of patients, we found that there was no correlation between the changes and prognosis for SNV or CNV alone or in combination. However, the differential expression of m1A-related writer, eraser, and reader m1A-regulatinggenes was analyzed separately in different T clinical stages. The results showed that overexpression of ALKBH1 was negatively correlated with clinical stages and good prognosis. Studies have also reported that the high expression of ALKBH3 was positively correlated with the advanced tumor stage of pancreatic cancer [37], which indicates that m1A does have clear prognostic value in pancreatic cancer. All this shows that demethylase provides a basis for the diagnosis and treatment of pancreatic cancer. However, the determination of specific prognostic markers requires further work. In 2016, Andrew Z Xiao’s team from Yale University first reported in Nature that ALKBH1 had DNA m6A demethylase activity. An increase of N(6)-methyladenine levels in Alkbh1-deficient cells leads to transcriptional silencing [38]. The reported substrates of ALKBH1 are different types of methylated nucleotides of DNA and RNA. ALKBH1 was shown to convert N6-mA into N6-hydroxymethyladenine (N6-hmA) *in vivo* [39]. In the aforementioned study of ALKBH5 in pancreatic cancer, same as an eraser gene, ALKBH5 overexpression inhibited the proliferation, migration and invasion of tumor cells. ALKBH5 regulates m6A demmethylation and reduces the mRNA degradation caused by YTHDF2 recognition of m6A, thus activating PER1 at the post-transcriptional level. The up-regulation of PER1 leads to the activation of ATM-CHK2-p53 / CDC25c signal pathway and inhibits cell growth [36]. In addition, it has been found that silencing ALKBH1 or HIF-1α can rescue the elevated level of MIAT (hypoxia response gene) in ox-LDL. Nuclear HIF-1α binds to the ALKBH1-demethylated MIAT promoter and up-regulates its transcriptional expression. Since ALKBH1 is a demethylase of m1A in mRNA, it is interesting to consider whether its function in cancers is related to its demethylation activity towards m1A in mRNA. Similar to ALKBH5, overexpression of demmethylase inhibits methylation activity, thereby regulating the expression of proto-oncogenes at the post-transcriptional level and inhibiting tumor progression. This is consistent with our findings in pancreatic cancer, indicating that ALKBH1 is a potential marker for the development of disease. In the present study, we identified its function and clinical importance in pancreatic cancer. All findings indicate that silencing ALKBH1 gene expression increases the growth and invasiveness of cancer cells. In contrast, overexpression of the ALKBH1 gene can induce apoptosis and inhibit cell proliferation [40]. We analyzed the Pearson correlation between m1A regulators and KEGG pathways and showed that the ErbB and mTOR signaling pathways were related to m1A methylation. Mammalian target of rapamycin (mTOR) is an important serine-threonine protein kinase downstream of PI3K/Akt. It can regulate the proliferation, survival, invasion, and metastasis of tumor cells by activating ribosomal kinase. This is consistent with the research results of Zhao et al. [33]. Therefore, we hope to further explore the specific role of the ALKBH1 gene in the regulation of downstream genes in the pathway. Cell proliferation and apoptotic pathways clearly play roles in the development of cancer [40]. However, the specific mechanism of action of the ALKBH1 gene has not been clearly reported, but it is a potential therapeutic target for a variety of cancers, such as gastric cancer [41], liver cancer [42], glioblastoma [43], and head and neck cancer [44]. It clearly has great clinical significance and therapeutic potential for cancer treatment.

In summary, we studied the genetic changes of m1A-regulating genes in pancreatic cancer and determined the functional expression levels of the ALKBH1 gene in GSEA, which confirmed its role in cancer development. We also found a prognostically valuable relationship between expression of ALKBH1 and the survival time of patients with pancreatic cancer. Since the role of m1A-regulating genes in pancreatic cancer has rarely been reported, especially the diagnostic basis of demethylase in tumors, this work provides new ideas for future study of the epigenetics of m1A in pancreatic cancer.

## Data Availability

All data generated or analyzed during this study are included in this article.

## Abbreviations

AUC: area under curve
CNV: copy number variation
FPKM: fragments per kilobase million
GSEA: gene set enrichment analysis
HIF-1α: hypoxia induced factor-α
LASSO: least absolute shrinkage and selection operator
m1A: methylation of N1 adenosine
m5C: 5-methylcytosine
m6A: methylation of N6 adenosine
PAAD: pancreatic adenocarcinoma
PDAC: pancreatic ductal adenocarcinoma
SNV: single nucleotide variants
TCGA: The Cancer Genome Atlas
TPM: transcripts per million
UTR: untranslated region
